# Obesity epidemic: impact from preconception to postpartum

**DOI:** 10.4155/fsoa-2016-0035

**Published:** 2016-08-19

**Authors:** Hind N Moussa, Mesk A Alrais, Mateo G Leon, Elizabeth L Abbas, Baha M Sibai

**Affiliations:** 1 Department of Obstetrics & Gynecology, University of Texas Health Science Center at Houston, Houston, TX, USA

**Keywords:** fetal programming, obesity, pregnancy, women

## Abstract

The obesity epidemic is on the rise throughout the USA and the world. Not only does it affect the general population but it also specifically poses unique threats to a woman’s life in the antepartum, peripartum and postpartum periods. An increased BMI is associated with worse perinatal outcomes, including higher rates of preeclampsia (and other hypertensive disorders), macrosomia, other neonatal morbidities and gestational diabetes. Isolated maternal obesity and additional maternal diabetes predispose the infant to potential adult disease through fetal programming. This review of the literature examines the effects of obesity on a woman’s life, outlining complications beginning with preconception through the postpartum period.

## Abstract (lay abstract)

**Lay abstract:** The obesity epidemic poses unique threats during the whole cycle of pregnancy. This review examines the effects of obesity on a woman’s life, outlining complications from prior to conception through the postpartum period. In addition, the contributions to adult disease of fetal exposure to this maternal risk are discussed. Future advances in nanotechnology and better understanding of placental function might allow more options in preventative and therapeutic interventions, yet with minimal fetal risk.

**Figure 1.**
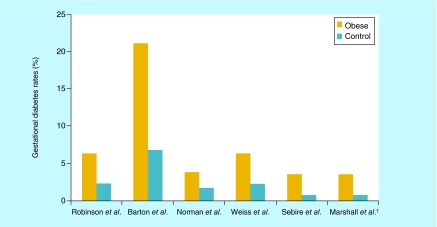
**Percentage of gestational diabetes comparing obese pregnant women versus control.** ^†^Comparison between super obese (BMI >50 kg/m^2^) vs obese (BMI 30–39.9 kg/m^2^).

**Figure 2.**
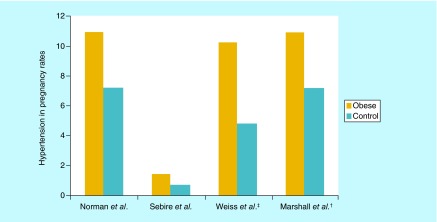
**Hypertensive disease in pregnancy in obese pregnant women versus control.** ^†^Comparison between super obese (BMI >50kg/m^2^) vs obese (BMI 30–39.9 kg/m^2^). ^‡^Included only gestational hypertension and not preeclampsia.

**Figure 3.**
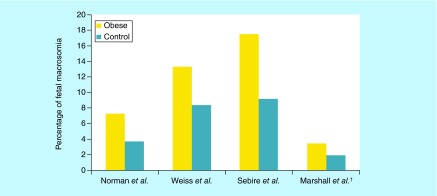
**Percentage of macrosomia comparing obese pregnant women versus control group within a population of study.** ^†^Comparison between super obese (BMI >50kg/m^2^) vs obese (BMI 30–39.9 kg/m^2^).

**Figure 4.**
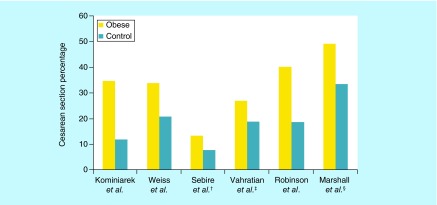
**Percentage of cesarean section comparing obese pregnant women versus control group within a population of study.** ^†^Included only emergent cesarean sections. ^‡^Included only primary emergent cesarean sections. ^§^Comparison between super obese (BMI >50 kg/m^2^) vs obese (BMI 30–39.9 kg/m^2^).

It is well known that the obesity epidemic is on the rise throughout the USA and the world. Not only does this epidemic affect the general population but also specifically poses unique threats to a woman’s life in the antepartum, peripartum and postpartum periods. According to the WHO, in 2014 more than 1.9 billion adults aged 18 and older were overweight of these over 600 million were obese with approximately 15% of women in the world considered obese [1]. Current estimates state nearly 65% of US adults are overweight or obese [1–4]. According to data from the NHANES study, the prevalence of obesity among women of reproductive age (20–39-year old) in the USA is nearly 31.8% [2]. Overweight and obese are defined as ‘abnormal or excessive fat accumulation that may impair health,’ and are measured by BMI of greater than 25 and 30 kg/m^2^, respectively [1].

With the rise in obesity also comes the rise of diabetes, which on its own also complicates the life of a childbearing woman. The prevalence of diabetes in 2010 was approximately 14% of the US adult population, which is projected to increase to 21% by 2050, though some estimates are as high as 33% [3]. This means the USA will move from the current rate of 1 in 10 adults having diabetes to nearly 1 in 3 by 2050 [3].

While obesity is a major risk factor for many health problems such as cardiovascular disease (CVD), diabetes, musculoskeletal disorders and some cancers [1], the risk it poses to a woman, in particular, has recently become a popular topic in research. This review of literature examines the effects and complications of obesity on a woman’s life, outlining complications beginning with egg quality through the postpartum period.

## Relevance

The alarming rise in obesity is relevant in that an increased BMI is associated with greater risks to the woman before, during and after pregnancy, as well as to her offspring. An increased BMI is associated with an increase in all studied perinatal outcomes, including preeclampsia (and other hypertensive disorders), macrosomia, other neonatal morbidities [5] and gestational diabetes. The incidence of gestational diabetes in obese pregnant women compared with the general obstetrical population is increased to 6–12 versus 2–4% [6,7].

### Preconception implications

While there is a clear association between obesity and subfertility, the subject is complicated by whether this association is due to elevated insulin levels impacting ovarian function (as many obese women have an increased prevalence of polycystic ovarian syndrome [PCOS]), or if obesity is wielding actual undesirable effects on the endometrium and ovulation. Multiple metabolic and endocrinologic origin etiologies explain this subfertility, as of obese women mature oocytes are of poorer quality than normal weight women [8], endometrial receptivity also plays a major role in the pathophysiology of their subfertility. Bellver *et al.* found that obese women receiving healthy oocytes from normal weight donors have lower rates of successful implantation than normal weight women [9], which might be related to different endometrial gene expression during the window of implantation in obese compared with control normal weight women [10]. Another factor to mention here is PCOS, obese women with PCOS have higher testosterone levels than lean women with PCOS, indicates a direct role of insulin signaling in the theca cells of the ovary to produce androgen and triggering a subsequent ovarian dysfunction [11].

Weight reduction in obese, infertile women correlates with an increased likelihood of pregnancy [12,13]. Preconception counseling is thus essential to educate patients about the influence of obesity on the outcome of pregnancy and to encourage them to maintain a healthy lifestyle and body habitus [14]. In 7 out of 11 studies systematically reviewed, the authors showed that preconception weight loss intervention will lead to significant increase in pregnancy rates in overweight or obese women undergoing Assisted Reproductive Technology [15]. However, weight reduction around the time of conception has its adversity also. If we look to the Dutch Famine that took place in WWII, which is considered as a good source for studies on maternal and fetal outcomes for preconception and early gestational undernutrition, we will find that, along with lower birth weights, these studies showed a long-term consequences such as a higher rates of glucose intolerance, atherogenic lipid profile, higher blood pressure rates, 3.2-fold increase in occurrence of microalbuminuria in adulthood, an increased prevalence of obstructive airways disease, coronary heart disease, schizophrenia and breast cancer [16–19]. In animal studies, preconception malnutrition or obesity appears to be related to the postnatal development of insulin resistance [20]. These changes might be explained as modifications that act as compensation in the immediate period to protect and prepare the offspring for an adverse environment [21].

One more point to mention here is fetal gender skew in obese women, the overall sex ratio in our population was close to 50:50, but individual mothers have a greater chance of bearing male offspring if their nutrient intake was high prior to conception [22].

### Pregnancy loss

A 2011 systematic review and meta-analysis that included 33 studies and nearly 48,000 *in vitro* fertilization treatment cycles revealed that in addition to the overall lower pregnancy and live birth rates, overweight and obese women also had considerably higher miscarriage rates compared with women with a normal BMI (<25 kg/m^2^) after treatment [23].

Another meta-analysis of 16 studies showed a general increase of miscarriage rates in women with a BMI greater than 25 kg/m^2^ versus normal weight females, regardless of the method of conception [24]. Another study revealed that overweight females did not have a greater embryo euploidy rate in first trimester miscarriages [25]. Other authors have concluded that because this increased risk of miscarriage in overweight females is independent of embryonic aneuploidy [24,26], perhaps the risk is because some obese women have PCOS or insulin resistance, which, as discussed earlier, is associated with a higher occurrence of pregnancy loss [14]. A 2010 retrospective case–control study that examined 204 miscarriages showed again that the excess risk of miscarriages in the overweight and obese population is independent of embryonic euploidy, and concluded that, while the mechanism remains uncertain and further studies need to be conducted to assess the impact of PCOS and insulin resistance in pregnancy outcomes, obesity is a risk factor for spontaneous miscarriage [24].

### Complications during pregnancy

Several studies have demonstrated increased perinatal complications with increasing maternal BMI. In 2012, a retrospective cohort study of over 64,000 births observed pregnancies of super obese (BMI >50 kg/m^2^) compared with obese women. The authors concluded that “Increasing maternal BMI was associated with a statistically significant increase in all studied perinatal outcomes, which included preeclampsia, macrosomia and composite neonatal morbidity, which included low Apgar score (<7 at 5 min), birth trauma, neonatal infection, neonatal hypoglycemia, respiratory distress syndrome, neonatal seizures, neonatal length of stay >5 days and/or meconium aspiration syndrome” [5]. Barton *et al.* showed that obesity in pregnant women age 40 or older had significantly higher rates of cesarean delivery, preeclampsia, gestational diabetes, preterm delivery and neonatal intensive care unit (NICU) admissions compared with nonobese women of the same age. In the same study, the rate of gestational hypertension and preeclampsia within the obese group was found to be higher in both younger and older women showing a strong association between hypertension and obesity [14].

Studies have shown that prepregnancy weight is well correlated to gestational weight gain, and assessing the latter seems to be relevant to the prevention of complications in obese pregnant women. Although behavioral interventions have been recommended to reduce the weight gain during pregnancy; however, the UPBEAT recruited 1555 pregnant obese woman (BMI >30) for randomized controlled trial, they randomly assigned participants to either a behavioral intervention or standard antenatal care, they found that behavioral intervention addressing diet and physical activity (in women with obesity during pregnancy) is not adequate to prevent gestational diabetes, or to reduce the incidence of large-for-gestational-age infants [27].

One of the major fetal complications in obese gravida is a stillbirth, which increases to 40% compared with nonobese gravida, with slight upward trend with increasing classes of obesity [28]. Other fetal side complication is congenital anomalies, the authors of a systematic review and meta-analysis showed that obese women are at increased risk of pregnancy complicated by neural tube defect, spina bifida, cardiovascular anomalies, cleft lip and palate, hydrocephaly and limb reduction anomaly; whereas the risk of gastroschisis was significantly reduced [28,29].

Complications highly associated with obesity include gestational diabetes mellitus, obstructive sleep apnea (OSA), limitations in ultrasound for fetal growth evaluation and fetal programming for adult disease, among others.

#### Diabetes mellitus

Obesity is a recognized risk factor for the development of both diabetes mellitus type II, and gestational diabetes [30]. However, many women who are first diagnosed with diabetes mellitus during pregnancy are classified as having gestational diabetes even though they have pre-existing diabetes that had gone undiagnosed. Pregestational diabetes and gestational diabetes mellitus (GDM) are two very different entities and distinction between the two is crucial [28].

##### Pregestational diabetes

Not only does diabetes has adverse effects on maternal health but it also has many effects on the fetus, from the immediate newborn period extending into the adult life. As with all cases of diabetes, blood sugar control is of utmost importance in directing health outcomes (as shown in a study by Reece and Homko, first-trimester HbA1C levels correlated directly with major malformations in the infant). Women whose HbA1C levels were greater than 12 had a 12-fold increase in major birth defects (Figure 1) [31].

Another study examined the association of fasting C-peptide, BMI and maternal glucose with the risk of preeclampsia. As the risk of preeclampsia is already higher in obese gravidas [5], this study also showed that there were strong, independent associations of higher fasting C-peptide levels and BMI with preeclampsia, regardless of maternal glucose levels [32].

Diabetes is also a risk factor for fetal macrosomia, which in itself can contribute to adverse pregnancy outcomes [33].

##### Gestational diabetes mellitus

In a large prospective multicenter database of more than 16,000 patients was studied, found that the obese and morbidly obese patients are at high risk for gestational diabetes compared with the control group [34].

Gestational diabetes is a condition in women who have carbohydrates intolerance with onset or recognition during pregnancy. It has been estimated that up to 6–7% of pregnancies are complicated by diabetes mellitus and that approximately 90% of these cases represent women with GDM [35]. Reece *et al.* mentioned that it is even higher, that this condition is most widespread in the USA with as many as 200,000 women, or 10%, of pregnancies being complicated by the illness every year [36]. An increased prevalence of GDM is found among Hispanic, Native American, Asian and Pacific Islander women.

Women with GDM are at higher risk of gestational hypertension (Figure 2), preeclampsia and Cesarean delivery but the most important is the increased risk of developing diabetes later in life. It is projected that up to 50% of women with GDM will develop diabetes 22–28 years after pregnancy that means a sevenfold increased risk of developing Type II diabetes mellitus (DM) in a woman with a history of GDM, compared with a woman without a history of GDM. This progression is influenced by ethnicity and the incidence of obesity [35].

#### Fetal macrosomia

Fetal macrosomia is another common complication among obese gravidas, both with and without diabetes. The American College of Obstetricians and Gynecologists (ACOG) defines macrosomia as infants weighing greater than 4500 g, though morbidity risks are still increased at more than 4000 g [37]. Macrosomia is more than two-times likely in women with a BMI greater than 30 kg/m^2^ [31], and the severity of macrosomia increases linearly with increasing maternal BMI. Macrosomia contributes to adverse maternal and fetal outcomes, including an increased risk of labor complications, birth injuries, and neonatal morbidity and mortality [38].

Macrocosmic infants have been shown to have more birth trauma, shoulder dystocia, higher death rates and lower Apgar scores. When delivered by cesarean section, macrocosmic infants had fewer birth injuries significantly, and the authors of several studies suggest delivering electively by cesarean if high-risk women screen positively for macrosomia [38,39]. One study, for example, showed that fetal macrosomia was associated with nearly a two-times increased the risk of an emergency cesarean section, longer maternal hospital stays and fourfold increased risk of shoulder dystocia. These infants also needed resuscitation and admission to the NICU more often than appropriately sized infants [38]. All of these studies point to the need for optimal planning and management for suspected macrocosmic pregnancies (Figure 3).

#### Fetal growth evaluation: limitation of ultrasound for fetal diagnosis

For many clinicians, performing an ultrasound examination of obese pregnant women proves to be more difficult than on nonobese women. Despite this, surprisingly few studies have been conducted regarding the limitation of ultrasound for fetal diagnosis and growth evaluation, even though the 20-week anomaly scan is one of the most important obstetric ultrasounds. One study demonstrated that once maternal BMI reaches higher than 90th percentile, fetal anatomy visualization rates fall by 14.5%, and concludes that BMI is the best predictor of visualization. No improvement in visualization was noted in regards to increased gestational age or examination time [40].

A more recent analysis examining sonography in overweight and obese women indicated that, despite 20 years of research in the field, the situation has not significantly changed, and ultrasound examinations still prove to be a difficult task in the obese gravida [41]. A recent retrospective cohort study of over 10,000 women evaluated the impact of maternal body characteristics on adequate visualization of fetal anatomy during the second trimester. The authors noted that visualization decreased significantly with increasing maternal BMI for most components of the anatomy scan and that the survey could only be completed 50% of the time during the first exam [42].

Despite the low number of studies, it is clear that obesity places limit on ultrasound evaluation of fetal growth and anatomy. In addition to the need for more research, there should also be a discussion with the obese gravida at the beginning of her pregnancy regarding the limitations and impact of obesity on ultrasound examinations.

#### Obstructive sleep apnea

Apart from the metabolic complications associated with obesity, there is also a well-known increased risk of sleep-disordered breathing patterns, particularly OSA. In pregnancy, physiologic alterations in the respiratory system, particularly those in the third trimester, contribute to an increased incidence of snoring and sleep-disordered breathing, which also reflects an increased incidence of OSA syndrome [43]. As obesity and pregnancy have both been studied to be significant and separate risk factors for breathing dysfunction, the obese gravid woman has been shown to have a significantly increased risk of sleep-disordered breathing in comparison to those of normal weight [44]. As OSA has previously been studied extensively in the nonpregnant patient and clearly linked to increased incidence of cardiovascular and cerebrovascular morbidity [45], it is of no surprise OSA in pregnancy has been under current research for its potential adverse effects on maternal and fetal health. An analysis of over 4000 women both with and without OSA examined the potential risk for adverse pregnancy outcomes, including low birth weight (LBW), preterm birth, small for gestational age (SGA), cesarean section, low Apgar score and preeclampsia [46]. The authors concluded that pregnant women with OSA were at a statistically significant increased risk for all of these complications when compared with those without OSA.

Some authors suggest that the current screening tools for OSA in nonpregnant patients may not be adequate for screening high-risk pregnant patients [47], and that alternative screening models should be considered due to the potential complications of OSA in pregnancy.

### Peripartum

#### Labor dystocia: abnormal labor patterns

Labor dystocia is a broad term that is more commonly categorized into several disorders, including both protraction and arrest disorders of the three stages of pregnancy. The first stage begins with the onset of painful contractions and cervical change up to the full 10 cm of dilation. The first stage is further divided into the latent and active phase, with the latent phase considered slow dilation up to 4 cm, and active phase as an increased rate of cervical change at 4–6 cm to full dilation. The second stage of labor begins with full dilation and continues through the delivery of the infant, and the third stage starts from the end of infant delivery through the delivery of the placenta. The first two stages may progress slower or faster depending if the mother is nulliparous or multiparous, respectively.

Several studies have linked increasing maternal BMI with an increased length of the first stage of labor [43,48–49]. In one large study of labor patterns in over 118,000 pregnant women, researchers found that total time to reach 10 cm increased as maternal BMI increased for both nulliparous and multiparas [49]. The authors suggest more time should be permitted for progress through the first stage of labor in these patients [43,49]. However, maternal obesity has not been independently associated with an increased duration of the second stage of labor [49–51].

Overall, due to the increasing incidence in overweight women of childbearing age, one must consider labor progression differences by maternal BMI before considering interventions through the first stage of labor, and perhaps consider redefining labor management protocols in this population [52–54].

#### Anesthesia concerns & complications

Physiologic changes to airway anatomy during pregnancy can contribute to an increased risk of intubation difficulties. One study using the modified Mallampati classification to assess maternal airways noted an increase in Mallampati score during pregnancy with increasing gestational age, with a 34% increase in class 4 airways at 38- versus 12-week gestation. The authors partially attribute this to increased weight gain and oropharyngeal edema [55].

However, obesity in the parturient also contributes to a myriad of increased anesthesia-related complications, more frequently than in nonobese women. These complications include higher rates of initial epidural failures, difficult or failed intubations [55–60] and increased multiple placement attempt failures [61]. Several authors suggest early placement of an epidural catheter to help prevent the need for general anesthesia, especially in the setting of an unplanned cesarean section, as is more often needed in the obese patient [56–58]. As is done in many facilities now, evaluation by an anesthesiologist before labor should be recommended for all obese pregnant patients because of the higher risk of complications in anesthetic management [56].

#### Increased C-section risk

Numerous studies and meta-analyses have been done that show increasing maternal pre-pregnancy obesity is associated with a significant increase risk of cesarean delivery – both elective and emergency – and decreased the incidence of vaginal deliveries [5,34,60–63]. Specifically, one meta-analysis estimated that the risk of a cesarean delivery was found to be more than double in obese women compared with women with a normal BMI (Figure 4) [59].

#### Effect on cardiovascular system

Obesity and pregnancy are associated with multiple physiologic changes, and many of these changes have similar implications, both conditions have profound effects on maternal cardiovascular system. Pregnancy is associated with a significant increase in cardiac output, which continues to rise until it reaches a level that is approximately 50% greater than that in the nonpregnant state. Obesity increases cardiac output even further because any extra amount of fat deposited in the body demands its share of cardiac output. Every 100 g of fat increases the cardiac output by 30–50 ml/min [56].

CVD is increasingly recognized as a frequent cause of pregnancy-related morbidity and mortality worldwide and contributed to nearly 12% of pregnancy-related deaths. The California Pregnancy-Associated Mortality Review examined a case series of 64 cardiovascular pregnancy-related deaths from 2002–2006; the authors found that one of the most prevalent underlying conditions among women who died from CVD was obesity (37.5%) [64].

### Postpartum

In addition to the numerous risks and complications in the ante- and peripartum period, obesity also poses several additional issues in the immediate postpartum period. Several studies have demonstrated that, when compared with nonobese women, obese patients had a higher prevalence of increased postpartum hospitalization stays, postpartum hemorrhage and infections, which contributed to overall increased healthcare costs [54,58,60–61,65–66]. As with many of the other complications mentioned above, increasing maternal BMI was associated with an increased magnitude of risk of postpartum complications [62].

The obese pregnant is also at a higher risk of having depression and anxiety symptoms, both antenatal and postnatal, than normal-weight pregnant [67].

#### Increased infectious morbidity

Numerous studies that together encompass hundreds of thousands of women have shown that, compared with women with a normal BMI, postpartum infection was considerably more common in obese pregnant women, independent of the form of delivery (vaginal, elective or emergency cesarean delivery), and despite the use of prophylactic antibiotics in the majority of studied cases [60,62,66]. Infections included wound, episiotomy and endometritis. As the authors of one study concluded, obesity is an independent risk factor for postcesarean infection [66]. This increased infection rate has been partially attributed to the altered metabolic state of obesity [62], as well as poor vascularity of subcutaneous adipose tissue and incision dehiscence [63,68]. Marrs *et al.* posed that vertical skin incisions in obese patients are associated with a lower risk of wound complications, however, no final conclusions have been yet achieved, and future randomized control trials are needed to answer this important clinical question [69].

#### Increased deep venous thrombosis/pulmonary embolus risks

The pregnant patient is already in a physiologic state of hypercoagulability, which predisposes her to increased risk of deep venous thrombosis and pulmonary embolism. Obesity is also a well-known independent risk factor for venous thromboembolism (VTE) [70]. Therefore, obese pregnant patients are at an appreciably increased risk of VTE during both the ante- and post-partum periods [60,70–71].

In addition to obesity, postpartum infection has also been found to be a significant risk factor for VTE in the obstetric patient [71], and since the rates of postpartum infections are increased in obese gravidas as noted previously, there are numerous mechanisms contributing to their overall increased risk of VTE, which altogether contributes to significant maternal morbidity and mortality [72].

### Long-term outcomes

The impact of maternal health on the future health outcomes of her child has been under study, since David Barker studied how nutrition and growth before birth altered the development of the heart. He demonstrated that newborns with a low birth weight were at a greater risk for developing coronary heart disease, a theory now well accepted as the ‘Barker Hypothesis’ [73].

Since the Barker Hypothesis studies began over 20 years ago, numerous studies have been done regarding the impact of maternal obesity during pregnancy. It has been shown in several studies that infants of overweight and obese mothers have significantly more fat mass at birth than infants born to mothers of normal BMI [74–78]. There is also shown to be a direct relationship between infant birth weight and BMI in later life [77,79]. While the authors admit that the data are preliminary in several of the studies, they suggest, similar to the Barker Hypothesis that the origins of future disease such as obesity and diabetes occur early in life [77]. Hence, maternal obesity and fetal macrosomia could predisposition infants to obesity later in life.

Several studies have been performed concerning alterations in the intrauterine environment predisposing to disease development later in life [80]. Gestational diabetes mellitus, along with pregestational diabetes, is a disease that metabolically impacts the fetal environment. Diabetes, as discussed previously, is linked with fetal macrosomia, which has independently been associated with obesity later in life. However, more studies have been conducted that link not only maternal diabetes but also isolated maternal obesity with future disease risk in the infant.

A study in the offspring of diabetic mothers found that alterations in the fetal and neonatal environment in the diabetic pregnant “…seem to program a disposition to develop obesity, diabetes mellitus and syndrome X-like alterations throughout later life.” [81]. Though the mechanisms of this connection are yet to be discovered, researchers admit to its complexity and have suggested numerous contributions such as alterations in hormones, insulin regulation, inflammation and metabolism, among others [49,51,80]. The author of one study suggested that hormones, when dysregulated in obese or diabetic gravidas, act as endogenous functional teratogens, and that early hyperinsulinism may lead to malprogramming of neuroendocrine systems regulating body weight, food intake and metabolism [80]. Once again, supporting the theory that the ultimate result is an increased disposition to obesity and diabetes later in life.

Due to the small and somewhat conflicting studies about the origins of obesity, a survey of over 14,000 children was conducted to examine the associations between birth weight, GDM and adolescent BMI. The authors concluded that higher birth weight and being born to a mother with GDM both increased the risk of being overweight in adolescence. However, this study claims that GDM may only be a risk factor, and not play a direct causal role in adolescent obesity since environmental factors in the postnatal period could also be a contributor to later obesity [51].

Another small study also supports this concept. Boney *et al.* found that children exposed to maternal obesity were at an increased risk of developing metabolic syndrome later in life, even if the mother was not diabetic. They suggest, similar to that of previous studies; obesity may still contribute to metabolic factors that impact fetal growth and postnatal outcomes [49].

While more research for the pathologic mechanism needs to be done, in animal studies they found that infants born to mothers fed a diet rich in fat, salt and sugar during gestation and lactation have higher daily energy intake than controls. Thus, weight gain and adiposity as a result of fetal exposure to a maternal obesogenic diet can be attributed to hyperphagia and higher preference for fatty food [82]. This hyperphagia might be explained by central insensitivity to leptin, the adipokine that is responsible for decrease food intake activity. This occurs due to prolong exposure to leptin *in utero* and increase its concentration in their peripheral circulation [83].

## Conclusion & future perspective

As can be seen from this literature review of just a selected number of studies in the field, countless research projects have been conducted on the impact of obesity on a woman’s life. Because of the impact it has on both the maternal and fetal life in the present and future, it is reasonable to suggest methods of possible intervention to address the obesity issue.

Several authors have recommended preconception counseling about weight loss before pregnancy, as women who can modestly lower their prepregnancy BMI decrease many of the previously discussed risks and adverse outcomes, including gestational diabetes mellitus, preeclampsia and macrosomia and risk for C-section [5]. As the recent ACOG committee opinion mentioned, counseling according to the medical risks that obesity represents to the patient is the responsibility of the physician, and this should be done in an unbiased manner, respecting both her autonomy, as well as her dignity [84]. Due to the alarming increased trend in obesity in the USA, however, this issue should be addressed from multiple disciplines, and not just left to the responsibility of the obstetrician. Primary care providers, communities, schools and employers can all make a significant contribution to raising awareness of the issue of obesity and assist in making changes toward a healthier lifestyle, as is being done in many places across the country.

Obese women who are planning to conceive should be encouraged to reduce their weight through diet, exercise, behavior modification and possibly bariatric surgery [85,86]. It is apparent that much more research needs to be done in the area of prevention, as well as optimizing recommendations for clinical practice, as the effect of providing antenatal interventions in overweight and obese pregnant women remains unclear [86,87].

Weight reduction around the time of pregnancy is still surrounded by uncertainty due to the lack of evidence and modest studies in this field. Behavioral, medical and surgical surgeries have shown some benefit to decrease complications as gestational diabetes and hypertension, especially with a wide margin between the intervention and pregnancy to reduce the effect of weight loss and undernutrition on the growing fetus. A large randomized control trial is needed to examine the best method to reduce weight in obese women during their reproductive years.

With this rising trend, we speculate that the cost of obesity will escalate to a level that we cannot afford to ignore both on the societal and psychological cost. Active steps should be taken in this field to modulate the effect of obesity in women, which might not eradicate the problem but considerably reduce the prevalence of this disease.

**Executive summary**

Obesity is one of today’s biggest health challenges in the USA, with a direct impact on women’s life throughout their reproductive years.
Preconception weight reduction is recommended to improve pregnancy outcomes, but when considering counseling obese women, it is important to highlight the fact that weight loss will neither guarantee pregnancy, nor will it guarantee a normal pregnancy outcome. Decision-making processes should be profoundly weighed to target the optimal outcomes in treating obese women who wish to conceive.
Obesity in pregnancy is associated with increased risk of pregnancy loss, gestational diabetes, gestational hypertension, fetal macrosomia, ultrasound limitations, stillbirth and obstructive sleep apnea.
At the time of delivery, obesity is related to more operative vaginal delivery, shoulder dystocia, higher risk of performing a cesarean section, surplus more complication during anesthesia.
During the puerperium, obesity will increase the risk of wound infection and thromboembolic events.
Recent research has found a link between maternal diet and offspring obesity as well as diabetes later in life, resulting in extended challenges to society.
Effective profound counseling for obese women during reproductive years should highlight all the risk factors, assess her unique medical situation and address the barriers to reduced weight.
Effort should be made to destigmatize obesity by adopting a nonjudgmental manner when counseling patients through prioritizing their autonomy and preserving their dignity.
More research is needed in the future to optimize recommendation and encourage prevention of this disease epidemic.
